# Lipoprotein-Associated Phospholipase A2 Activity as Potential Biomarker of Vascular Dementia

**DOI:** 10.3390/antiox12030597

**Published:** 2023-02-28

**Authors:** Giovanni Zuliani, Judit Marsillach, Alessandro Trentini, Valentina Rosta, Carlo Cervellati

**Affiliations:** 1 Department of Translational Medicine and for Romagna, University of Ferrara, Via Luigi Borsari 46, 44121 Ferrara, Italy; 2 Department of Environmental & Occupational Health Sciences, University of Washington, 4225 NE Roosevelt Way, Seattle, WA 98105, USA; 3 Department of Environmental and Prevention Sciences, University of Ferrara, Via Luigi Borsari 46, 44121 Ferrara, Italy

**Keywords:** Lipoprotein-associated phospholipase A2, β-Amyloid, biomarkers, blood, neurological disorders

## Abstract

A wealth of evidence suggests that Lipoprotein-associated phospholipase A2 (Lp-PLA2) plays a relevant role in atherogenesis and inflammation, which in turn are associated with the risk of developing dementia. The aim of this study was to evaluate whether serum Lp-PLA2 activity might be an early and/or late biomarker for different forms of dementia. Serum Lp-PLA2 activity was assessed in older patients with mild cognitive impairment (MCI, *n* = 166; median clinical follow-up = 29 months), Late-Onset Alzheimer’s disease (LOAD, *n* = 176), vascular dementia (VAD, *n* = 43), dementia characterized by an overlap between LOAD and VAD (AD-VAD MIXED dementia) (*n* = 136), other dementia subtypes (*n* = 45), and cognitively normal controls (*n* = 151). We found a significant trend towards higher levels of Lp-PLA2 activity in VAD compared with the other groups (ANOVA, *p* = 0.028). Similarly, Lp-PLA2 activity was greater in MCI converting to VAD compared with those that did not or did convert to the other types of dementia (ANOVA, *p* = 0.011). After adjusting for potential confounders, high levels of Lp-PLA2 activity were associated with the diagnosis of VAD (O.R. = 2.38, 95% C.I. = 1.06–5.10), but not with other types of dementia. Our data suggest that increased serum Lp-PLA2 activity may represent a potential biomarker for the diagnosis of VAD.

## 1. Introduction

Lipoprotein-associated phospholipase A2 (Lp-PLA2) has been widely suggested to be an independent predictor of cardiovascular disease (CVD) [1,2,3]. This enzyme is a calcium-independent PLA2 belonging to group VII, mainly secreted by macrophages and platelets, and circulates in the blood in association with low-density lipoprotein (LDL), and, to a lesser extent, high-density lipoprotein (HDL) [4]. It has been suggested that the net effect of Lp-PLA2 action depends on the carrier, antioxidant, or pro-inflammatory if it is associated with HDL or LDL particles, respectively [1,4,5].

Lp-PLA2 hydrolyzes the acetyl group at the sn-2 position of platelet-activating factor (PAF, indeed, it is also named PAF acetylhydrolase) thereby inactivating this pro-inflammatory phospholipid (PL). It is also capable to degrade oxidized PL (antioxidant activity) with a chemical structure similar to that of its natural endogen substrate to generate lysophosphatidylcholine and oxidized fatty acids, which have pro-inflammatory properties [4,6]. In turn, these by-products are believed to mediate the onset and progression of the inflammatory response in atherogenesis [4,6].

There is a wealth of evidence showing that Lp-PLA2 mass and activity are predictors of CVD and stroke [3,7]. It is well-known that inflammation, atherogenesis, and more in general, cardiometabolic risk factors and cerebrovascular disease are also associated with dementia incidence [8,9,10]. This was the rationale of a few observational studies exploring the potential link between Lp-PLA2 and cognitive impairment. The reported findings were inconclusive, with some showing increased serum/plasma levels of these enzymes in patients with dementia [11,12,13,14], and others failing to disclose significant alterations [15,16,17]. In particular, it remains unclear whether Lp-PLA2 could be an early biomarker of dementia and whether it could discriminate between different forms of this syndrome. To shed light on this uncertain scenario, we assessed Lp-PLA2 activity in a large sample including patients with mild cognitive impairment (MCI), Late-Onset Alzheimer’s disease (LOAD), Vascular dementia (VAD), dementia characterized by an overlap between LOAD and VAD (AD-VAD MIXED), other less frequent types of dementia and cognitively healthy controls.

## 2. Materials and Methods

### 2.1. Subjects

Seven hundred and seventy subjects referring to the Center for Cognitive Decline and Dementia (CDCD) (University of Ferrara, Ferrara, Italy) were enrolled in the study as detailed elsewhere [18,19]. The samples included:

- One hundred sixty-six amnestic MCI patients, defined as those with a presence of either short or long-term memory impairment, with or without impairment in other single or multiple cognitive domains. Most of these individuals were affected by amnestic multi-domain MCI. The classification of these subjects is based on the absence of dementia, according to the standardized criteria for this syndrome [20]. Subjects with MCI caused by documented conditions or diseases (for example, major depression, and severe vitamin B-12 deficiency) were excluded from the study. These patients were consecutively enrolled after the first visit and MCI diagnosis at the CDCD; they underwent a regular clinical follow-up as outpatients (mean = 29 months; range = 11–156) (see major detail in [21]). The Mini-Mental State Examination (MMSE) ranged from 18 to 28 (median = 24.6/30). Seventy-eight enrolled (55%) progressed to dementia within the follow-up period.
- One hundred seventy-six patients with Late-Onset Alzheimer’s disease (LOAD), with a disease onset after the age of 65. The diagnosis was drawn according to the National Institute on Aging–Alzheimer’s Association (NIA-AA) workgroups criteria [22]. Following these well-established clinical criteria, we included only patients with “probable” LOAD, while patients with “possible” LOAD or with LOAD and cerebrovascular disease were not excluded. Mini-Mental State Examination (MMSE) ranged between 18–23 and the Clinical Dementia Rating (CDR) ranged between 1–4.
- Forty-three patients with the diagnosis of VAD according to the National Institute of Neurological Disorders and Stroke and Association Internationale pour la Recherché et l’Enseignement en Neurosciences (NINDS-AIREN) criteria for a diagnosis of probable VaD [23]. The initial diagnosis of VAD was confirmed by magnetic resonance in all cases. The subjects had an MMSE ranging between 19–23.
- One hundred thirty-six with MIXED AD-VAD; in these patients, a definite diagnosis of LOAD or VAD was not possible since they presented both the characteristics of VAD (e.g., significant vascular disease, focal neurological signs) and LOAD (e.g., memory impairment, type of progression). MMSE ranged between 16–23; CDR ranged between 1–2.
- Forty-five patients with other forms of dementia (Dementia subtypes: Lewy Body disease/Parkinson’s dementia, frontotemporal dementia, condition related to psychiatric conditions, neoplasm/metastasis, hydrocephalus, Fahr’s syndrome, alcohol-related, not defined). MMSE ranged between 18–25; CDR ranged between 1–2.
- One hundred fifty-one cognitively healthy subjects (Controls). Subjects enrolled in this group complained of no memory problems and did not present symptoms of cognitive impairment or any related functional disabilities. MMSE ranged between 26–30.

All subjects included were informed about the research project and gave their written consent before being included in the study. For people with dementia, relatives or caregivers were asked to sign the informed consent form. The study was carried out according to the Declaration of Helsinki (World Medical Association, http://www.wma.net accessed on 25 July 2017), and the guidelines for Good Clinical Practice (European Medicines Agency, http://www.ema.europa.eu accessed on 25 July 2017). The institutional review board of the University of Ferrara approved the study (study n. 170579).

The diagnosis of dementia was drawn by trained geriatricians. Personal data and medical history were collected through a structured interview with patients and caregivers. All patients underwent a general and neurological examination. All enrolled patients underwent neuropsychological evaluation by a standardized battery of tests including MMSE, Rey’s 15 words, Raven progressive test, clock drawing test, and routine clinical tests for the evaluation of agnosia, apraxia, and aphasia. Assessment of functional disabilities was made by Instrumental Activities of Daily Living (IADL) and Basic Activity of Daily Living (BADL). Routine clinical chemistry tests in blood were performed to exclude other causes of cognitive impairment. Subjects affected by severe congestive heart failure, severe liver or kidney disease, severe chronic obstructive pulmonary disease, and cancer were excluded. The use of non-steroidal anti-inflammatory drugs, antibiotics, or steroids led to the exclusion from the study.

### 2.2. Biochemical Parameters

Ten mL of venous blood was sampled from the antecubital vein using a 21-gauge and collected into regular serum-separating tube vacutainers with a clot activator. Blood sampling was performed in the morning with patients in a sitting position. Every patient was required to be in a fasting state. After 30 min at room temperature, the samples were centrifuged at 4650× *g* for 20 min at 4 °C and serum was separated and stored in single-use aliquots at −80 °C until analysis. The levels of plasma lipids (total cholesterol, HDL-cholesterol, and triglycerides), albumin, and high-sensitivity c-reactive protein (hs-CRP) were assessed by the centralized laboratory of Sant’Anna Hospital (Ferrara) by standard enzymatic techniques. Levels of LDL-cholesterol (LDL-C) were obtained according to Friedewald’s formula.

### 2.3. Lp-PLA2 Activity Assay

Serum activity levels of Lp-PLA2 were assessed by using 2-thio PAF as substrate by a spectrophotometric assay (by using Tecan infinite M200 Tecan Group Ltd., Männedorf, Switzerland). The substrate of Lp-PLA2, thio PAF (Cayman Chemical, Ann Arbor, MI, USA), was first resuspended in ethanol and then subdivided into aliquots which were finally placed at −80 °C. On the day of the assay, the solvent in the substrate aliquot was evaporated under a stream of nitrogen, for approximately 2 min. Subsequently, thio-PAF was resuspended in assay buffer (containing: 100 mM Tris, 0.1 mmol/L Ethylene glycol-bis(2-aminoethylether)-N,N,N′, N′-tetraacetic acid (EGTA), pH = 7.2) to a final concentration of 400 nM. Ten microliters of serum were mixed with 5 μL of assay buffer and 10 μL of Ellman’s reagent (containing, 5,5′-dithiobis-(2-nitrobenzoic acid, DTNB). This mixture was incubated at room temperature in dark conditions for 30 min. The reaction was initiated by adding 200 μL of substrate solution and followed for 10 min. The formation of free thiols, due to the catalyzed hydrolysis of thio PAF, was detected according to Ellman’s procedure, as described in Ref. [24]. A molar extinction coefficient of 13,600 M^−1^ cm^−1^ (wavelength = 410 nm) was used for the calculation of enzyme activity, as expressed in units per liter (U/L).

### 2.4. Statistical Analyses

The normal distribution of all continuous variables was first checked by the Shapiro-Wilk test. According to the outcome of this statistical test, the levels of normally distributed variables were expressed as mean ± standard deviation (SD), or median (interquartile range) when they were non-normally distributed. Parametric and non-parametric analyses were employed according to the variable distribution. *T*-test and one-way analysis of variance (ANOVA), with Bonferroni correction for multiple comparisons, were used to compare two and more than two groups of subjects, respectively. Analysis of covariance (ANCOVA) was made to check the effect of potential confounding factors on the outcome variable. Non-parametric Mann–Whitney U test and Kruskal-Wallis were employed to compare medians. The comparison of the prevalence of categoric variables were assessed by the χ^2^ test. Correlations between continuous variables were analyzed by Pearson’s and Spearman’s tests. Multivariable logistic regression analysis (using the levels of Lp-PLA2 activity below the median calculated in controls as cut-off) was performed to evaluate the effect of selected covariates on the relationship between Lp-PLA2 and different forms of dementia. All statistical analyses were carried out using SPSS for Windows statistical package, version 13.0.

## 3. Results

### 3.1. Demographic and Main Clinical Characteristics of the Population Sample

Table 1 displays the general characteristics of the sample subjects. Healthy controls were the youngest group. The difference was statistically significant (*p* < 0.001 for all -Bonferroni post hoc tests) with all groups with the exception of dementia subtypes. Female gender was less prevalent in Controls and MCI patients (around 50%) compared with the other study groups (range 64–70%) (*p* < 0.001, for all posthoc comparisons). Regarding comorbidities, the prevalence of CVD and diabetes did not significantly vary among the groups. Hypertension was more prevalent in VAD patients (*p* < 0.01 compared with Controls). No significant changes in lipid profile, albumin, and Hs-CRP were detected across the subject groups. As expected, years of formal education, and scores of neuropsychiatric and functional tests (MMSE, IADLs, and BADLs) were significantly higher in Controls compared with all the other groups (*p* < 0.001 for all).

### 3.2. Serum Lp-PLA2 Activity in Controls, MCI, and Patients with Dementia: Cross-Sectional Analysis

**Figure 1:**
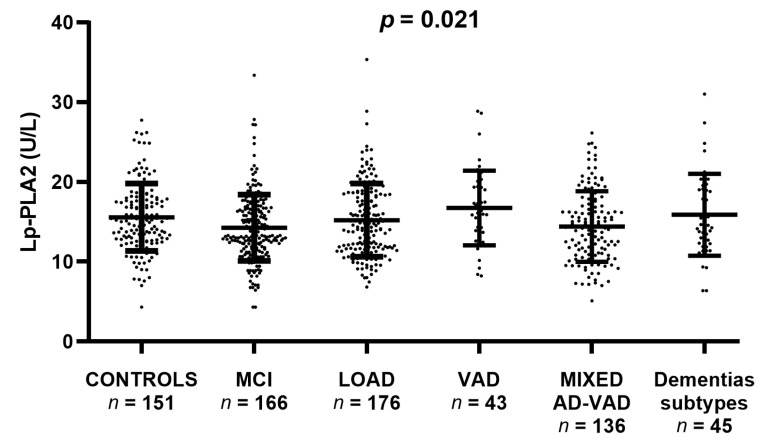
Serum Lp-PLA2 activity levels in Controls, Mild Cognitive Impairment (MCI), Late-Onset Alzheimer’s Disease (LOAD), Vascular Dementia (VAD), AD-VAD MIXED dementia, and other dementia subtypes. Comparison between groups was performed by ANOVA which yielded *p* = 0.021.

Serum Lp-PLA2 activity was different across the study groups as highlighted by ANOVA *p* = 0.021). In particular, the most evident differences were the increase of enzyme activity in VAD compared with Controls (+12%) and of the latter compared with MIXED AD-VAD (+18%). On the contrary, MCI, LOAD, and other dementia subtypes showed median values similar to Controls (Figure 1).

As a second step, we evaluated the possible influence that covariates might have on the association between Lp-PLA2 and the diagnosis of LOAD, MCI, VAD, or MIXED AD-VAD. We found that LDL-C and HDL-C were positively and inversely correlated with enzyme activity (r = 0.351 and *p* < 0.0001 and r = −0.259, *p* < 0.001, respectively; Supplementary Figure S1A,B). Notably, Lp-PLA2 did not correlate with age (r = 0.011, *p* = 0.779, Supplementary Figure S2). Moreover, a further comparison after the exclusion of younger Controls confirmed the lack of influence of this variable on Lp-PLA2 (ANOVA, *p* = 0.036, Supplementary Table S1).

The activity of Lp-PLA2 was significantly higher in men compared with women (*p* < 0.001, Supplementary Figure S3) and in individuals affected by CVD and diabetes mellitus (*p* < 0.05 for both, Supplementary Figures S4 and S5, respectively). Of note, the difference in Lp-PLA2 between men and women retained its significance after adjusting for potential confounders such as age, LDL-C, and HDL-C (*p* < 0.001). On the contrary, the change observed in subjects with CVD and diabetes disappeared after adjusting for these confounding factors.

### 3.3. Serum Lp-PLA2 Activity in Converter and Non-Converter MCI: Longitudinal Data

**Figure 2:**
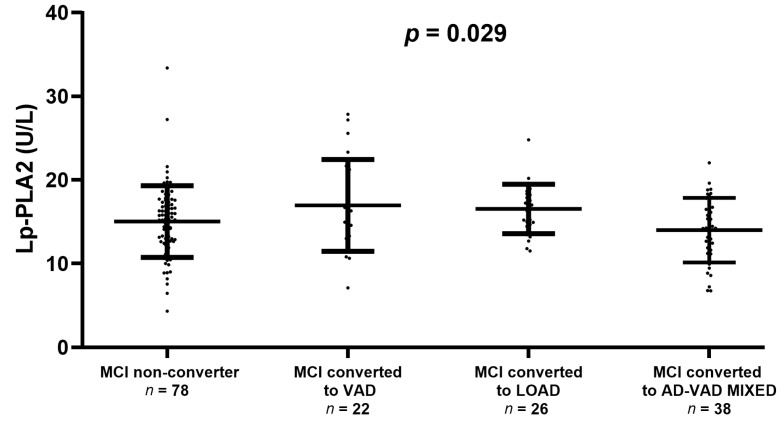
Serum Lp-PLA2 activity levels in Mild Cognitive Impairment (MCI) that remains stable (MCI non-converter) or converting to Vascular Dementia (VAD), Late-Onset Alzheimer’s Disease (LOAD), or AD-VAD MIXED. The plot does not display the levels of Lp-PLA2 in MCI converted to other dementia subtypes because of the very low number of subjects (*n* = 2). Comparison between groups was performed by ANOVA which yielded *p* = 0.029.

We also checked whether Lp-PLA2 activity might be associated with the progression from MCI to overt dementia (Figure 2). There was no significant difference in enzyme activity between the converter and non-converter (15.0 ± 4.2 U/L and 15.5 ± 4.0 U/L, respectively). However, we found a significant trend towards higher levels of Lp-PLA2 in MCI converted to VAD or LOAD compared with stable MCI (ANOVA, *p* = 0.029, Figure 2).

### 3.4. Odds of Having MCI, AD, VAD, and AD-VAD MIXED

**Figure 3:**
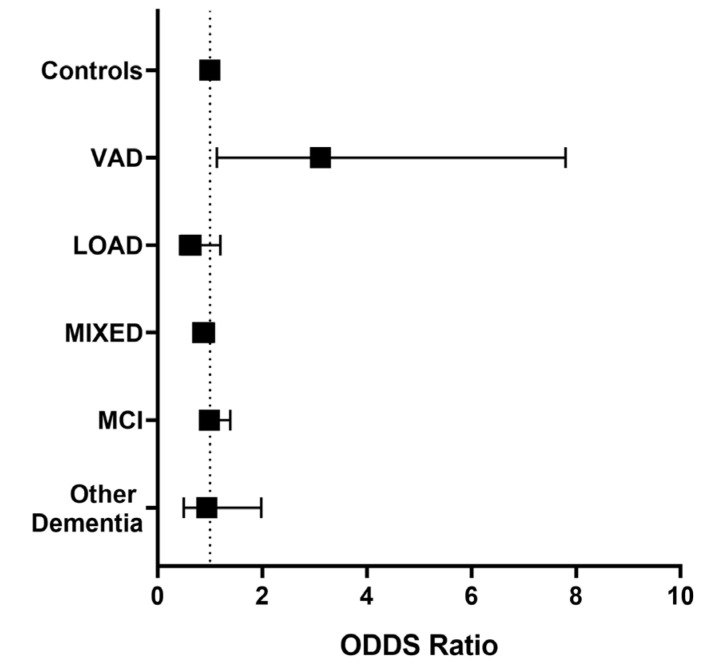
Adjusted odds ratios (95% C.I.) for diagnosis of Mild Cognitive Impairment (MCI), Late-Onset Alzheimer’s Disease (LOAD), Vascular Dementia (VAD), AD-VAD MIXED dementia, and other dementia subtypes compared with controls in individuals with high levels of Lp-PLA2 activity levels (≥15.2 U/L). Covariates: LDL-C, HDL-C, sex, age, diabetes, and CVD. The analysis was performed by multivariate logistic regression; significant OR for VAD diagnosis (O.R. = 3.115, 95% C.I. = 1.13–7.98).

Since a trend toward higher levels of Lp-PLA2 activity was observed in VAD compared with the other groups included in the study, we evaluated whether an Lp-PLA2 activity higher than the median value (15.2 U/L) might be associated with the risk of being affected by dementia or MCI compared with Controls, after controlling for possible confounders. As disclosed in Figure 3, higher Lp-PLA2 activity levels were associated only with a higher likelihood of receiving a VAD diagnosis compared with Controls (O.R. = 3.115, 95% C.I. = 1.13–7.98), after adjustment for LDL-C, HDL-C, age, sex, diabetes, and CVD.

Finally, we checked whether increased Lp-PLA2 activity could discriminate among the different types of dementia. We found that higher levels of these enzymes were associated with greater odds of being affected by VAD compared with LOAD (O.R. = 2.76, 95%C.I. = 1.20–6.30), AD-VAD MIXED (O.R. = 3.50, 95%C.I. = 1.51–8.01), other dementia subtypes (O.R. = 2.38, 95%C.I. = 1.03–5), but not to MCI (O.R. = 2.38, 95% C.I. = 0.67–4.839).

## 4. Discussion

The present is the first study dealing with the cross-sectional and longitudinal evaluation of serum Lp-PLA2 activity, a marker of atherogenesis, in individuals with different types of dementia or MCI. The main finding is that higher levels of Lp-PLA2 are significantly associated with VAD diagnosis, and the association is independent of potential confounders including LDL-C, HDL-C, sex, age, diabetes, and CVD. This is important since these are both some of the main risk factors for VAD and LOAD and strong predictors of Lp-PLA2 levels [7,8,25]. Remarkably, from the clinical point of view, higher levels of this enzyme activity appear to be able to discriminate VAD from other forms of dementia.

On the contrary, Lp-PLA2 activity was not significantly increased in MCI patients and failed to predict the conversion to dementia. However, we observed a significant trend towards higher levels of phospholipase activity in MCI converted to VAD compared with MCI stable or converted to other types of dementia.

The relationship between Lp-PLA2 and dementia has been already investigated in a few studies and the findings were inconclusive. The first and largest observational study (*n* = 6713 people), was published in 2006 and showed that healthy subjects in the highest quartile of Lp-PLA2 levels activity were at higher risk of all-cause dementia. However, the relationship was weak and the lack of measurement of LDL-C might have affected the accuracy of the findings. Indeed, as we demonstrated, this lipid parameter is strongly correlated with Lp-PLA2, and thus, it should be taken into account as a covariate in multivariate analysis [13]. 

In line with these findings, the Cardiovascular Health Study, including 5888 community-dwelling older adults, found an increased risk of developing AD in patients with low levels of Lp-PLA2 mass [14]. This large prospective study also found a strong association between increased Lp-PLA2 activity and the diagnosis of MIXED AD-VAD. Notably, these results were independent of confounding factors such as APOE ε4 allele, vascular comorbidities, inflammation markers, and lipid profile (although HDL-C was not assessed and thus not included in the multivariate analysis). An increase in Lp-PLA2 in AD was also reported in two studies with cross-sectional design [26,27]. In contrast, Davidson et al. [16], detected no significant differences in Lp-PLA2 activity between AD, aMCI, and control subjects. Additionally, the authors reported no significant correlation of Lp-PLA2 with cerebrospinal fluid (CSF) biomarkers of AD (Aβ42, t-TAU, and p-TAU), nor with white matter changes. Similar results were obtained by the Framingham Study, which explored the relationship between some conventional and unconventional CVD risk factors, AD, and all-cause dementia, demonstrating an overlap with those for cardiovascular disease. From this longitudinal study, it emerged that plasma concentration of Lp-PLA2 was not significantly associated with the risk of developing dementia or AD [17].

In the above-cited investigation, Lp-PLA2 is described as a biomarker of inflammation, which plays a critical role in the onset and progress of atherosclerosis. The involvement of this lipoprotein-associated enzyme as a proactive agent in atherogenic and inflammatory processes is the major rationale for our and other studies on its association with dementia.

Indeed, there is a wide consensus that atherosclerosis and low-grade inflammation are, although often subclinical, conditions associated with cognitive decline in the elderly [28,29,30,31,32]. High Lp-PLA2 reflects in enhanced phospholipid hydrolysis, high contents of oxidized non-esterified fatty acids and PLs are produced, which promote expression of adhesion molecules, stimulate cytokine production (TNF-α, IL-6), and attract macrophages to the arterial intima [33,34,35]. This exacerbates endothelial dysfunction and accelerates the growth of the plaque and, eventually, the formation of a necrotic core [14]. Owing to the direct role in atherosclerosis and the widely documented association between circulatory levels and CVD prevalence, the trend towards greater levels of Lp-PLA2 in VAD compared with the other dementias, was not surprising, but even not obvious. Indeed, it is well-established that VAD, the second most common form of dementia after AD, has a preponderant vascular component, being characterized by the presence of matter lesions or hyperintensities, and macro- and micro-cerebral infarcts [36,37,38]. It is also true that “neuro-vasculopathy” is also a common feature of AD and this, along with the overlap of symptoms and risk factors, makes the differential diagnosis challenging [9,39]. Our multivariate data suggest that Lp-PLA2 may help as a potential biomarker for discriminating between the two diseases, with this ability apparently not influenced by classical cardiometabolic risk factors such as dyslipidemia, diabetes, and history of previous CVD [40,41,42].

A similar trend shown in the cross-sectional analysis also emerged from longitudinal-like evaluations of the association between Lp-PLA2 activity levels and the progression to dementia in MCI patients. This category of patient is of paramount importance in clinical investigations of dementia. Individuals with MCI are more likely to progress to dementia (15% per year), as compared with non-amnestic forms [43]. For this reason, MCI still represents the main target population for pharmacological trials on AD. Our results are novel and intriguing; indeed, they suggest that an alteration in Lp-PLA2 may precede the development of VAD. However, we are aware that our findings should be corroborated by studies on a larger sample, with sequential measurements of Lp-PLA2 at different time points.

A further interesting result of the present investigation was the sexual dimorphism of Lp-PLA2. A similar increase in men compared with women was reported by a number of other large population-based studies [25,44,45]. Of note, to the best of our knowledge, only one of these studies (Dallas Heart Study, *n* = 3332), Ref. [25], adjusted the analysis for both LDL-C and HDL-C levels. This is important since these two parameters (which we included as covariates) have a strong association with Lp-PLA2 levels. The possible explanation of this observed phenomenon is the down-regulatory effect of estrogens on Lp-PLA2 expression [46].

Finally, we would wish to underline other important limitations of this study. The main caveat of the study is the lack of CSF biomarkers assessment; thus, the misclassification of some patients cannot be ruled out. The unavailability of CSF inevitably affects the clinical relevance of our findings. However, these biomarkers are employed for the diagnostic confirmation of AD, not VAD (i.e., the only form of dementia that we found to be associated with a change in Lp-PLA2). It should be noted that the NINDS-AIREN criteria (which state that evidence of vascular disease on magnetic resonance imaging for the brain is mandatory) for probable VAD have a low sensitivity (about 20–60%) but a high specificity (about 90–99%) as reported by clinical and neuropathological studies [23,47,48]. Thus, while we might have a non-negligible number of false negatives (i.e., individuals with VAD included in other dementia groups), the number of false positives would be very low. In this regard, it appears unlikely that the finding of an elevated serum Lp-PLA2 in VAD is unreliable due to the lack of CSF biomarker confirmation. Second, we cannot exclude that biases or unmeasured confounding factors (primarily obesity) might also influence the development of the forms of dementia considered in the study. However, we took into account a number of potential confounders, and the observed increase in Lp-PLA2 activity was independent of those factors. Finally, due to the reported association between pro-inflammatory cytokines and VAD [49], their assessment could add valuable data, other than potential covariates to include in the multivariate analysis.

## 5. Conclusions

We found that serum Lp-PLA2 activity increases in VAD. We also showed that this biomarker is able to discriminate this form of dementia from LOAD. This is an important novel finding because there are still no available fluid biomarkers to use for a differential diagnosis between VAD and LOAD. Further larger studies employing validated CSF biomarkers are warranted to confirm these findings.

## Figures and Tables

**Table 1:** Main characteristics of the sample according to diagnosis.

	CONTROLS (*n* = 151)	MCI (*n* = 166)	LOAD (*n* = 176)	VAD (*n* = 43)	MIXED AD-VAD (*n* = 136)	DEMENTIA SUBTYPES (*n* = 45)
**Age (years)**	75 ± 6	78 ± 5 ^a^	80 ± 5 ^a^	80 ± 6 ^a^	79 ± 4 ^a^	77 ± 6
**Female gender (%)**	53	54	70 ^a,b^	65 ^a,b^	65 ^a,b^	64 ^a,b^
**Formal Education (years)**	8 (5–13)	5 (5–8) ^a^	5 (3–7) ^a^	5 (3–5) ^a^	5 (5–6) ^a^	5 (5–8) ^a^
**MMSE score (/30)**	27 (25–29)	25 (23–26)	20 ^a^ (18–23)	21 ^a^ (18–23)	20 ^a^ (17–23)	22 ^a^ (19–25)
**-Current smoker (%)**	7	9	7	10	7	2
**Comorbidities**						
**-Hypertension (%)**	57	63	64	70 ^a^	64	58
**-Diabetes (%)**	13	15	14	19	15	20
**-CVD (%)**	10	15	12	14	17	18
**Functional status**						
**-IADLs**	6 (5–8)	7 (4–8)	3 (1–5) ^a^	3 (2–4) ^a^	3 (1–6) ^a^	4 (1–6) ^a^
**-BADLs**	6 (5–6)	6 (5–6)	5 (4–6)	5 (4–5)	5 (4–6)	5 (3–6)
**Clinical chemistry**						
**Tot-Chol (mg/dL)**	206 ± 41	205 ± 39	210 ± 41	211 ± 42	205 ± 48	204 ± 30
**LDL-C (mg/dL)**	124 ± 36.24	122 ± 34	126 ± 35	126 ± 36	120 ± 42	123 ± 26
**Triglycerides (mg/dL)**	102 (82–132)	98 (76–133)	93 (75–132)	111 (77–178)	108 (85–140)	98 (69–121)
**HDL-C (mg/dL)**	60 (47–71)	61 (49–71)	62 (57–74)	57 (45–71)	58 (49–70)	59 (50–69)
**Albumin (g/dL)**	3.9 (3.8–4.3)	4 (3.8–4.2)	3.9 (3.8–4.2)	3.9 (3.7–4.3)	3.9 (3.8–4.1)	3.9 (3.8–4.1)
**Hs-CRP (mg/dL)**	0.2 (0.1–0.3)	0.1 (0.1–0.3)	0.2 (0.1–0.4)	0.3 (0.1–0.7)	0.2 (0.1–0.4)	0.1 (0.0–0.4)

Continuous variables are expressed as mean ± SEM or median (interquartile range). Categorical variables are expressed as percentages within groups. Abbreviations: CVD, cardiovascular diseases; MMSE, Mini-Mental State Examination; LOAD, Late-Onset Alzheimer’s Disease; IADLs, Instrumental Activities of Daily Living; BADLs, Basic Activity of Daily Living. Post-hoc test: ^a^ *p* < 0.05 vs. controls; ^b^ *p* < 0.05 vs. MCI.

## Data Availability

Raw data will be made available on request.

## Supplementary Materials

The following supporting information can be downloaded at: https://www.mdpi.com/article/10.3390/antiox12030597/s1, Figure S1: Correlation between serum Lp-PLA2 activity levels and LDL-C (A) and HDL-C (B); Figure S2: Correlation between serum Lp-PLA2 activity levels and age; Figure S3: Correlation between serum Lp-PLA2 activity levels and sex; Figure S4: Correlation between serum Lp-PLA2 activity levels in healthy individuals and patients with CVD; Figure S5: Correlation between serum Lp-PLA2 activity levels in healthy individuals and patients with diabetes. Table S1: Levels of serum Lp-PLA2 activity in Controls, MCI, AD, VAD, MIXED AD-VAD, and Dementia subtypes after exclusion of younger Controls.
