# Supplementary material for: Lipoprotein-Associated Phospholipase A2 Activity as Potential Biomarker of Vascular Dementia
Source: Antioxidants (Basel). 2023 Feb 28;12(3):597. doi: 10.3390/antiox12030597 (PMC10045550; doi:10.3390/antiox12030597)
Supplement: Supplementary file 1 [file antioxidants-12-00597-s001.zip › antioxidants-2220926-supplementary.pdf]

SUPPLEMENTARY MATERIALS

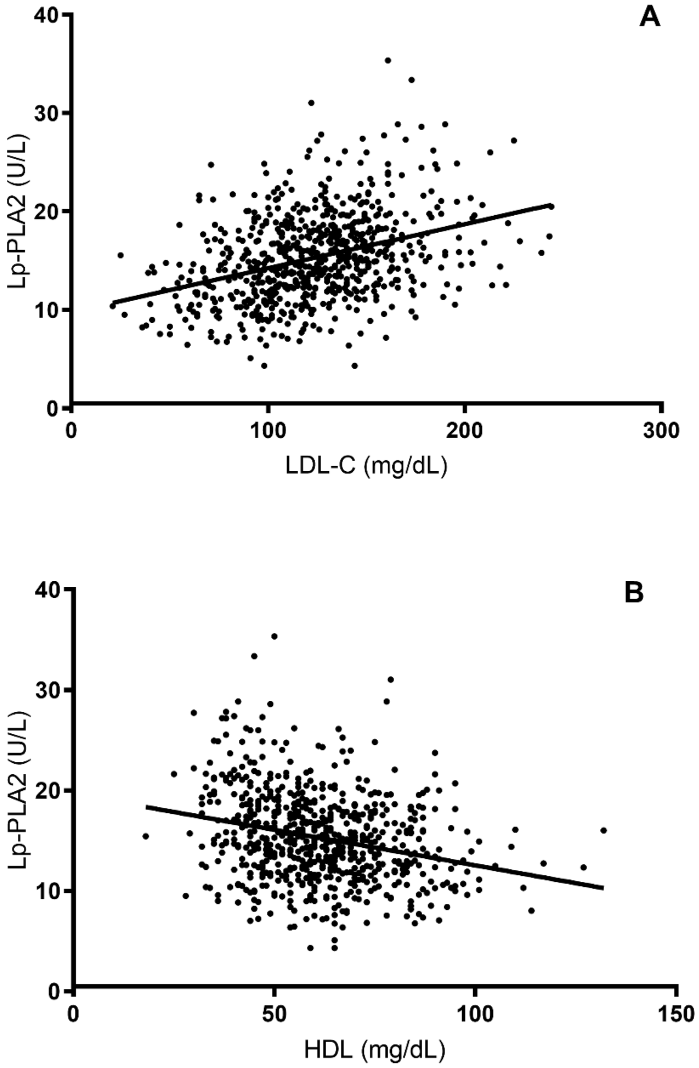

Figure S1. Correlation between serum Lp-PLA2 activity levels and LDL-C (A) and HDL-C (B).

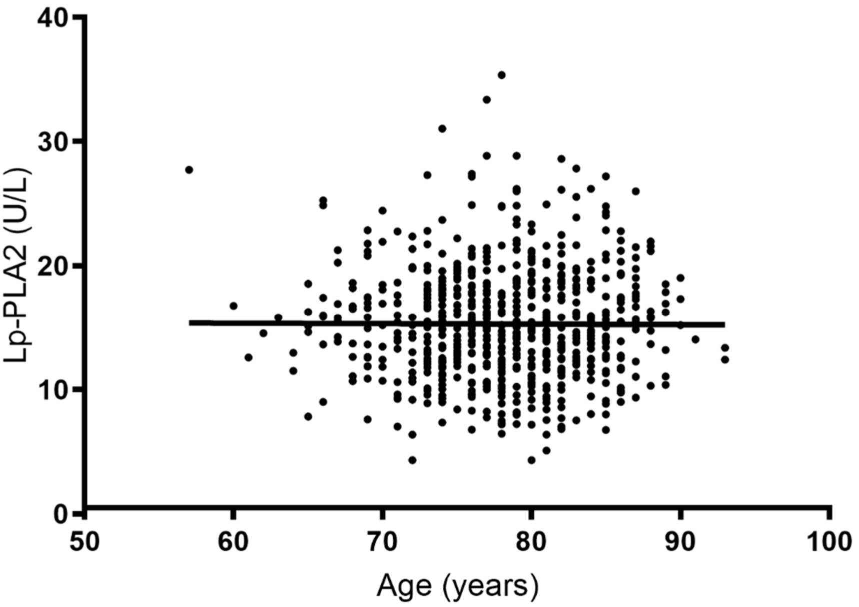

Figure S2. Correlation between serum Lp-PLA2 activity levels and age.

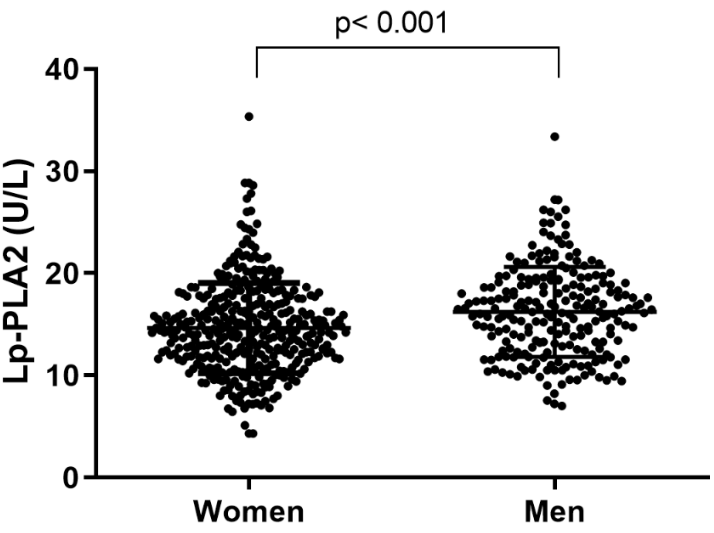

Figure S3. Correlation between serum Lp-PLA2 activity levels and sex.

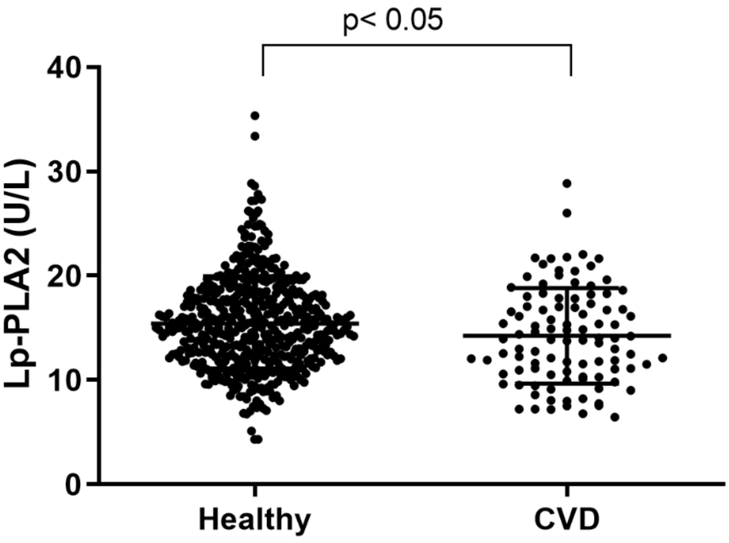

Figure S4. Correlation between serum Lp-PLA2 activity levels in healthy individuals and patients with CVD.

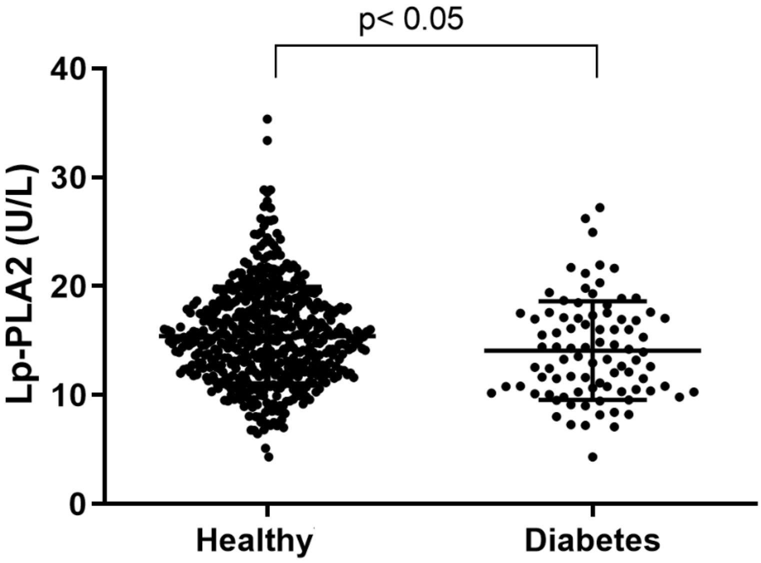

Figure S5. Correlation between serum Lp-PLA2 activity levels in healthy individuals and patients with diabetes.

Supplementary Table 1. Levels of serum Lp-PLA2 activity in Controls, MCI, AD, VAD, MIXED AD-VAD, and Dementia subtypes after exclusion of younger Controls.

	**CONTROLS (n:86)**	**AD (n:177)**	**MCI (n:164)**	**VAD (n:43)**	**MIXED AD-VAD (n:138)**	**DEMENTIA SUBTYPES (n:45)**	**ANOVA (P)**
**Lp-PLA2, U/L**	15 ± 4	15 ± 4	15 ± 4	17 ± 4	14 ± 4	15 ± 4	0.036
**Age, years**	80 ± 4	80 ± 5	78 ± 5	80 ± 6	79 ± 4	77 ± 6	0.101

Continuous variables are expressed as mean ± SEM.
